# Regular Meeting of the Fulton County Medical Society, July 1, 1915

**Published:** 1915-07

**Authors:** Allen H. Bunce


### REGULAR MEETING OF THE FULTON COUNTY MEDICAL SOCIETY, JULY 1, 1915.

By Allen H. Bunce, M. D.

Dr. M. B. Hutchins exhibited a case showing extensive skin-grafting after removal of the breast for adeno-carcinoma. A clinical diagnosis of adeno-carinoma was made and the Rodman operation was followed in removing the breast. No enlarged glands were encountered. In attempting to close the wound after the operation a space of three or four square inches was left uncovered by skin. This having failed to unite, skin-grafting was done seventeen days later. Novocaine and adrenalin were used as a local anesthetic and the skin for the graft was obtained from the thigh. A razor blade held in an artery forceps was used to shave off the graft, much care being used not to cut too deep. All the raw surface was covered except a small area at one point. Four days later all the grafts had taken and the wound was entirely covered except the area not covered with the graft originally. Dr. Hutchins said that the points of interest in this case were first, the rather extensive area covered and, second the rapid growth and perfect result obtained from the grafts.

Dr. J. S. Derr exhibited a number of interesting X-Ray plates, among which were the following:

I. Normal chest.
II. Calcified tuberculosis of the lungs which had been overlooked upon physicial examination.
III. Tuberculosis infiltration of the left lung and a part of the right.
IV. Tuberculosis of the right lung.
V. Tuberculosis of the lungs with cavity formation.
VI. Tuberculosis of the lung with compression by nitrogen. gen.

Dr. Derr was thanked for this exhibit as a contribution to the symposium of the evening by the president on behalf of the Society.

Next the papers of the evening which constituted a "Symposium on Tuberculosis," were read. These appear elsewhere in this issue of the Journal. They were:

I. Gross Pathology.—Dr. John Funke.

II. Objective Symptoms and Subjective Symptoms.—Dr. L. C. Roughlin.

III. Sputum.—Dr. J. E. Paullin.

IV. Inspection, Palpation, Percussion.—Dr. H. L. Reynolds.

V. Auscultation.—Dr. Arch Elkin.

VI. Treatment.—Dr. E. C. Thrash.

Dr. W. T. Jones discussed the importance of rheumatism and of a slight rise in the temperature as two of the earliest symptoms in many cases of tuberculosis. In making examinations of applicants for insurance he has been able to detect tuberculosis in many by a systematic examination and record of the temperature. Dyspepsia should never be passed by unnoticed as it is often one of the earliest symptoms. Chronic appendicitis and gall-stones may sometimes be hard to differentiate from a beginning pulmonary tuberculosis from the symptoms alone. In doubtful cases the X-Ray should always be made use of as it is one of our most reliable aids in doubtful cases. Finally, a careful microscopical and chemical examination of the sputum should be made when possible.

Dr. A. F. Quillian said he agreed with Dr. Rouglin on the importance of a slight rise in the temperature and an accelerated pulse. However, in cases of the chronic fibroid type we often have a normal temperature and a normal pulse. Here we are forced to rely on a careful physical examination. In pulmonary hemorrhage Dr. Quillian reports favorable results from the use of emetine. He considers the tonsils an important point of entrance for the tubercle bacilli. The most important single factor in the diagnosis of a suspected case of pulmonary tuberculosis is the physical examination. A perfected technique and a proper understanding of the physical signs can be relied upon in all cases.

Dr. C. H. Paine said that the papers presented had covered the ground in the diagnosis and treatment of pulmonary tuberculosis as well as it could be done in the length of time alloted to the subject. There was nothing further which he cared to add.

Dr. M. G. Campbell stressed the importance of the X-Ray in revealing the presence of enlarged mediastinal glands. As these glands are often the seat of the tuberculosis process, the knowledge that they are enlarged enables the physician to begin treatment at once and probably prevent future trouble. Here Dr. Campbell asked the opinion of the society as to the value of the Moro Test in the diagnosis of a doubtful case. He considers the treatment with drugs as being entirely unsatisfactory. The only logical treatment is for each case to be put in a sanatorium as soon as a diagnosis is made. The usual sanatorium case does not remain in the sanatorium for a sufficient length of time. He thinks each case should remain there for at least a period of two years. After they have recovered sufficiently they should be allowed to work under the direction of the physician in charge of the institution.

Dr. R. R. Daly said the subject of the reflex origin of cough had interested him for some time. This, he considered an important point in the differential diagnosis of tuberculosis. Recently he read a paper on the subject of reflex origin of cough in which he reported two typical cases. Both of these cases, men at the ages of 23 and 36 respectively, exhibited irregularities in the throat which caused very persistent and aggravated cough with pain in the chest. One of the cases gave a history of hemorrhage. Upon examination he found enlarged and irritated lingual tonsils which, when removed, relieved the trouble immediately.

Dr. M. T. Davis thought there was much of value in what had been said regarding the diagnosis and treatment of this important disease. He emphasized the importance of a careful studying every case carefully. He regards the appearance of fine rales and an increase of vocal fremitus to the whispered voice as important diagnostic points. A low blood pressure with accelerated pulse and a rise in temperature he considers significant and call for a further examination. Tubercle bacilli may or may not be present in the sputum in an early case. However, an increaes of the cell content and the alubumin is almost universally found. An increase in the small mononuclears above 30 per cent and an increase in the amount of albumin in the sputum are almost pathognomonic of tuberculosis. Dr. Davis reported five of his private cases in which the latter statement was true.

Dr. O. O. Fanning emphasized the importance of coming in daily contact with a large number of cases of tuberculosis in order to be able to diagnose it properly. He said such an opportunity was offered at the Atlanta Anti-Tuberculosis Association where he and a number of other physicians held clinics daily. He said the two most important things were, first, to find if the patient has tuberculosis and, second, to prevent him from giving it to others. This can be done only by educating the physician as to a correct diagnosis and the patient as to the proper care of himself.

Dr. A. H. Lindorme reported finding a considerable percentage of men who applied for enlistment in the National Guard to be suffering with tuberculosis. Here he rejects men upon finding roughened vesicular breathing and a prolonged expiratory murmur. The subsequent history of a number of men has justified this course.

Dr. W. N. Adkins reported some interesting experiments which had been carried out by the late Dr. VanGiesen with creosote and lime salts.

Dr. C. W. Gould reported an autopsy at the Grady Hospital in which he found a tuberculous pneumonia, engrafted on an old chronic case of tuberculosis, where both lungs were entirely consolidated. He thought it remarkable that the man should have been able to be up until the very day he died, he having dropped dead at the Terminal Station.

In closing, Dr. Elkin said he disagreed with Dr. Davis who said rales were necessary for the early diagnosis of a case as tuberculosis. Dr. Elkin said rales were not at all necessary to diagnose an incipient case. He answered the question of Dr. Campbell in regard to the Moro Test by saying that he considered it of no value whatever.

Dr. Thrash said in closing that he would like to put his remarks under three heads, viz:

1. The absence of tubercle bacilli in the sputum is of no value whatever in excluding tuberculosis. The temperature is one of the most important factors to be considered.

2. Cough is not necessery. To wait for the appearance of a cough is to lose valuable time.

3. In the treatment of hemorrhage:

(1) Keep the patient quiet in bed. Use morphia if necessary to control cough.

(2) Lower blood pressure by magnesium sulphate. Don't depend upon nitroglycerine as it's effect is too transitory.

(3) Increase the coagulability of the blood by the use of horse serum.

The local tests for tuberculosis are of no value.

---

### A Letter from Dr. Pruitt.

Several weeks ago, Dr. M. C. Pruitt left his practice in Atlanta to go to London and to engage in the medical service of the Allies. His equipment here for such work was unquestioned and his enthusiasm made him seem to us a valuable addition to any Staff that would accept his offered services. He writes to us that the way was not so clear as he expected but that the obstacles have been removed and his work has commenced. The letter which hints at more than he thought he could say because of censorship brings us into closer touch with the situation and is given herewith:

Westminster Hospital,
Broad Sanctuary S. W.
July 29, 1915.

My Dear Doctor:

I have been expecting to write to you for several days or weeks, but I have been a very busy man until Friday last.

On arriving in England, I learned that it was impossible for one to do any work other than that of Assistant House Surgeon or something similar because of the British Medical Act, which requires British qualifications. I investigated the matter and found that the only possible way to get on the British Registrar was to have a degree from either Irish, English or Scottish Medical colleges. This was "some pill," but I made up my mind to see how much of my work would be accepted and how long it would take. I made application to the Royal College of Physicians and Surgeons of Edinburgh and was admitted to the finals. The examination was held at Glasgow. I am enclosing a clipping from the Edinburgh paper as to the results and the degrees granted.

Most of my time has been spent in the Royal Infirmary at Edinburgh, but I am now in London.

Yesterday I accepted the place as House Surgeon in Westminster Hospital and started at work to-day. Just how long I shall stay here depends upon other appointments.

As to the medical question and the war: There are plenty of Doctors at the present time to handle the situation; doubtless they will need more later. France is much more in need than England but each place has work provided one can unravel the vast amount of red tape which comes between the applicant and the appointment. However one can readily see that it is a time for this country to be careful about everything they do regardless of how small it may be. It is most interesting to work out these problems.

London is the same old London and one can not tell by a superficial view that she is in a state of war. The external evidence of the feminine gender in the business world is becoming more and more evident and such places as porters, street car conductors and ticket collectors are being filled by women. Regarding women and shell making, Mrs. Pankhurst says that an ordinary, intelligent woman can learn to do such skilled work in three weeks.

As to the duration of the war no one can tell now what will be done, but the end looks a good way off to me. The spirit is good and the effort is better, so I can see but one result and that is in favor of the Allies. I am sending you one of my photographs which was made at the time I received my degrees from the Royal College of Surgeons at Edinburgh.

With regards. Fraternally,

M. C. PRUITT.

